# Assessing Rolling Abilities in Primary School Children: Physical Education Specialists vs. Generalists

**DOI:** 10.3390/ijerph17238803

**Published:** 2020-11-26

**Authors:** Pietro Luigi Invernizzi, Gabriele Signorini, Dario Colella, Gaetano Raiola, Andrea Bosio, Raffaele Scurati

**Affiliations:** 1 Department of Biomedical Sciences for Health, Università degli Studi di Milano, 20129 Milan, Italy; pietro.invernizzi1@unimi.it (P.L.I.); gabriele.signorini@unimi.it (G.S.); 2 Department of Clinical and Experimental Medicine, University of Foggia, 71122 Foggia, Italy; dario.colella@unifg.it; 3 Department of Human, Philosophical and Education Sciences, University of Salerno, 84084 Fisciano (SA), Italy; graiola@unisa.it; 4 Human Performance Laboratory, Mapei Sport Research Centre, 21057 Olgiate Olona (VA), Italy; andrea.bosio@mapeisport.it

**Keywords:** interpretation, perception, competent professional, practice, didactics by competence, proficiency barrier, reflective teaching styles, consciousness

## Abstract

Teaching physical education requires competencies to conduct the classes and to assess the motor skills of practitioners. Specialists (physical education professionals) and generalists (primary school teachers) differently experienced motor tasks during their academic education. This study aimed to compare the teachers’ ability in assessing the children’s forward and backward rolls from the analysis of the reliability of an evaluation grid of rolling abilities (Information Scale for Agility on the Soil, InfoSAS), which was investigated in a first study with teachers. A second study in young children explored the responsiveness of the InfoSAS to discriminate by skill level or by training effects. When administered by specialists, the InfoSAS resulted in being reliable (forward: *p* = 0.087 and *p* = 0.908; backward: *p* = 0.926 and *p* = 0.910; intra- and inter-rater reliability, respectively) and responsive in detecting differences due to expertise (gymnasts vs. primary school children; forward: *p* = 0.003, backward: *p* = 0.016) or improvements after specific training in rolling (pre- vs. post-children’s training; forward: *p* = 0.005, backward: *p* = 0.001). The results support the conclusion that specialists exhibit higher competence than generalists, which allows proper application of the InfoSAS, possibly because of the practice of skills and reflective teaching styles in physical activity they experienced, along with their academic education in sport sciences.

## 1. Introduction

Competence is a pivotal concept of the teaching process at any school level. It can be defined as the individual capacity/ability to deal with job, study, professional, or personal requirements by applying all knowledge and skills previously acquired in formal, non-formal, and informal learning contests [1]. Knowledge has to be turned into practice, which moves the focus of the teachers’ training from individual knowledge to operative skills. Nevertheless, theory and practice are not independent features of teaching competence; they have to be integrated to connect “knowledge”, “know-how”, and “know how to make to-do” among them. To allow this, attention should be given to the working and learning methods the teachers must manage. Therefore, the focus of the teachers’ training should not be limited to considering the acquisition of knowledge or planning contents, exercises, and lesson units, but should also include considering the acquisition of the most suitable methods and teaching styles to deal with any situational constraints.

From this perspective, the curricula of the degree courses in physical education (PE) are aimed to train proficient professionals to be skilled in finding appropriate solutions to apply to any educational scenarios they are facing by best summarizing what they have learned and what, especially, they have experienced during the instruction process, and by problem solving and didactical laboratory experiences in particular [2,3]. Practical experiences and reflective actions with classmates and professors allow future physical educators to fully realize and comprehend the learners’ perceptions and troubles in motor control, and to select the proper teaching strategies to be employed while leading physical activity. Thanks to an integrated approach of the curriculum of study (i.e., integrating cognitive, emotive, and social competencies), PE professionals acquire the soft skills (SS) [4] that add to the European key competencies (EKC, the educational recommendations from the European Community) [5], citizenship competencies (CC) [6], and objectives of the specific competencies (OC). The latter includes specific skills to evaluate the purposes of PE, which allows PE professionals to achieve SS, EKC, and CC, and become fully competent [7].

**Figure A1:**
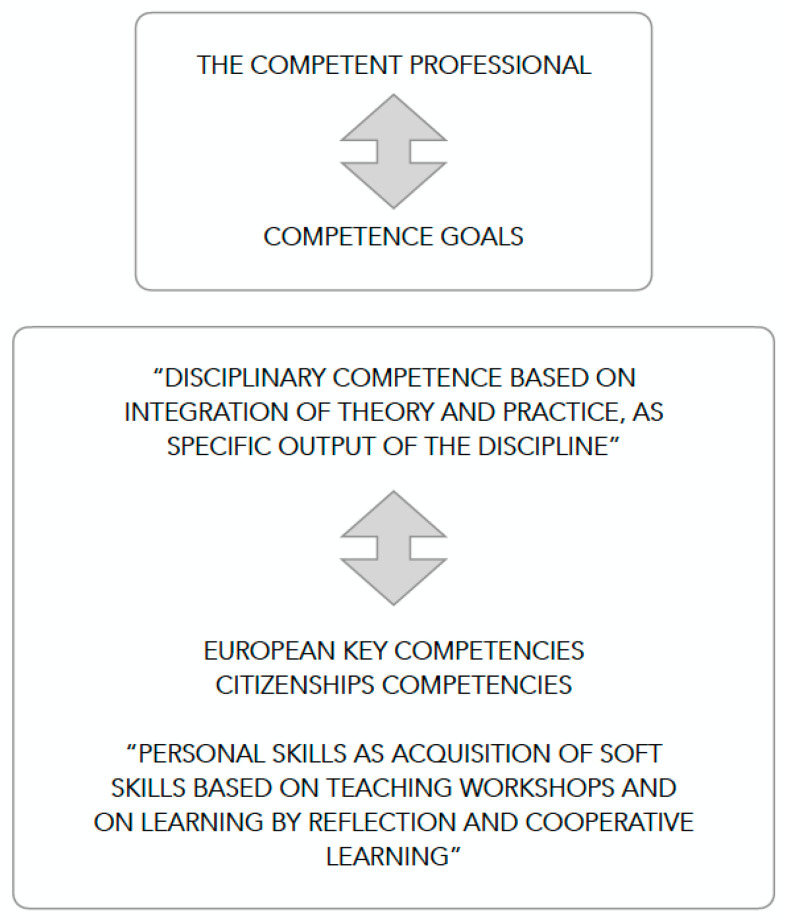
Guidelines to the structure and planning of the curriculum to train a competent professional in physical education.

**Figure A2:**
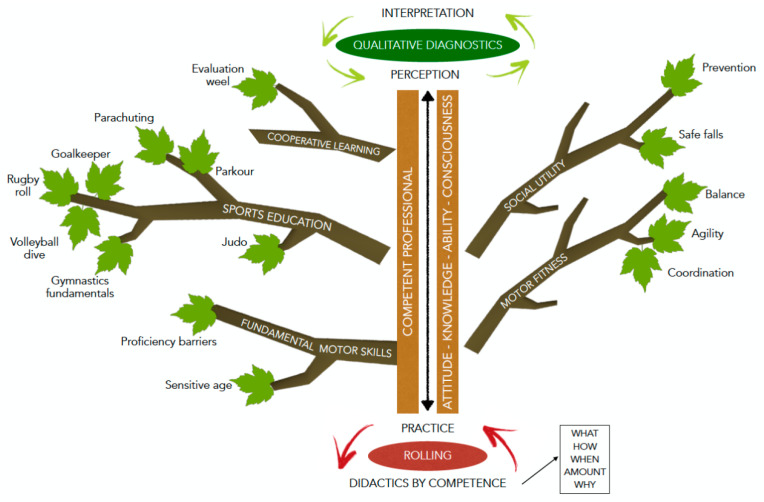
Allegorical representation of the features of a proficient PE teacher: “The Tree of Competence”. As in a tree, the main features of a proficient PE teacher are displayed: the skills of a competent professional (the log of the tree) allow proper management of the tasks to be learned/trained and their goals (the branches), thanks to the acquired competence to interpret/evaluate their outcomes (the top), which is strengthened by individual experiences and consciousness (the roots).

To prepare a competent professional, the structure and planning of the curriculum should be set by considering the acquisition of different competences (Appendix A, Figure A1 and Figure A2) to shift the “education to the movement” into the “education through the movement” [7,8,9]. The assessment of motor competencies to be transmitted to the learners requires the teacher to be able to collect and analyze the outcomes of the observed motor activities, and to detect all connections and relationships involved. Previous examination, experience, evaluation, and attention on the motor activity to be taught is therefore required, possibly by applying the methodology of the “wheel of evaluation”, consisting of self-evaluation and reflexive procedures and starting from forms which define the key features to teach, learn, and improve a task [10], and by experiencing cooperative learning [11].

A further issue to be considered is how task observation can be performed. Anyone perceives and pays attention differently to what she/he considers to be relevant to better complete a task, further interpreting in her/his own way what has been seen [12]. For this reason, the observation process has to be thoroughly envisaged and mastered from the teacher, and tools to make as objective as possible the observation and the evaluation of the learners’ skills should be set. In a previous study, it was suggested that specialists (PE specialists) better approached teaching PE compared to generalists (teachers of primary school) by Invernizzi, et al. [13]. Their competency in evaluating the motor tasks possibly differed because of personal experience in physical activity, which has been previously practiced in particular throughout the study course [14].

The teacher’s attitude in approaching and assessing the rolling abilities in young children served in this study to compare specialists’ and generalists’ approaches while leading physical activity of primary school children. Rolling abilities were selected, as rolls and rotations on the transverse axis are frequent in gymnastics and in basic motor skills involved in improving fundamental motor skills (FMS), and are also very useful in several other sports or everyday life [15,16]. In particular, forward or backward rolls require (and likewise enhance) strength and agility, and further improve the use of sensory cues, which makes gymnastics skills extremely advantageous to consolidate and improve balance and postural control in children [17]. It is noteworthy that an improvement in FMS begins from the control of movement stability and evolves in the development of movement mobility [18]. It has been claimed that the control of movement stability is more species-typical (innate and more consistent across humans). For this reason, a child can develop stability strategies without specific support [19]. In many motor skills, the stability strategy makes the child “freeze” joint movement (to simplify the movement control), limiting the capability and flexibility in responding to perturbations that challenge the correct execution of the skill [20]. During a roll on the ground, which is a static-dynamic FMS that evolved from crawling, the contact with the soil strengthens the self-perception and consciousness of the body segments that otherwise are often hardly perceived [16]. A good dynamic balance acquired thanks to rolling proficiencies increases the child’s ability to participate in a variety of sports, further contributing to developing skills, leading to lifelong physical activity, and acting as falling prevention [21,22].

Practice based only on non-structured free play during childhood is not enough to determine the improvement of FMS [23]. Guiding the child through instructions and structured practice to improve his ability and make him overpass the proficiency barrier is therefore paramount during childhood. The education on rolling patterns should be set in childhood when children are most responsive to FMS acquisition [24,25]. Postponing the learning of rolling patterns to adolescence may lead to less effective results for the learners because of their different ways to cope with the fear and uncertainty of the body’s control, especially in unusual rollovers and, more than likely, to a proficiency barrier that occurs between the fundamental and transitional levels of motor skill development. Children who reach high levels of competence above this proficiency barrier are more skilled than ones who have delayed motor experiences, and they are more like to continue engaging in physical activity throughout their lifespan [23,26]. Similarly, even postponing the learning of the rollover skills can be dangerous, especially for overweight children who experience troubles in the biomechanical execution of rolls because of their morphological features [27] or their limited muscular strength and ankle, hip, and back flexibility that are required to appropriately complete a roll [28,29], possibly causing unsafe conditions for the cervical spine [30].

Hence, learning forward and backward rolls in childhood appear to be suitable to enhance the sensory-motor system and motor control, to prevent injuries, to overcome proficiency barriers, and definitely to improve in particular the FMS and agility on the soil, promoting motor fitness [31] and lifelong physical activity [32]. Rolling patterns and agility on the soil allow learning how to protect one’s body during the ground impact, increasing the chance of avoiding or minimizing the effects of collisions with the ground. Indeed, people practicing fighting sports such as judo rarely get injured, despite the high number of falls that happen, possibly because of their great motor abilities, technical skills, and, of course, proficiency in the rolling patterns acquired [33]. Nevertheless, the “Ukemi”, in which a rolling on a transverse axis is executed by isolating the cervical zone, is one of judo’s fundamentals [34].

The design of this study has been based on the hypothesis that the learning process and the “conceptual maps” coming from connections of multiple theoretical and practical experiences as depicted in Figure A2 (Appendix A) may support the training of students in PE to better understand all structural requirements of specific topics, such as rolling abilities, and to achieve the most adequate level of comprehension and interpretation of the motor patterns to be applied in situational contexts [35,36]. Based on the self-determination theory applied to the teachers, learning and transferring teaching competences to different contexts are fostered by the environment, intended as the cultural carrier and vessel of human interactions, and by motivation as the source of will to activate and take actions [37,38]. Therefore, educational academic plans that use methodologies, such as practical experiences and reflection on and by actions to enhance competence, autonomy, and relationships, are global experiences that help to facilitate the integrated involvement of every domain of personality (motor, cognitive, and social), and better contribute to training motivated, mindful, and competent professionals. Therefore, through exploration of the reliability and responsiveness of an easy but accurate instrument to evaluate the rolling abilities (i.e., agility on the soil) in young children, this study aimed to compare specialist and generalist teachers during the application of the assessment tool in physical education classes. We hypothesized that specialists, thanks to their practical background based on reflective teaching styles, have full competencies to properly apply the instrument to assess and evaluate the performance of learners, while generalists more scarcely manage physical activity and its evaluation, despite their wide general knowledge in didactics for the specific age of the participants involved in the study.

## 2. Materials and Methods

The study was performed under the principles of the 1975 Declaration of Helsinki and was approved by the local Ethical Committee (Nr. 34/18).

After a full explanation of the purpose of the study, all participants or parents/legal tutors signed a written informed consent before the study. They were allowed to withdraw from the study at any time. The reliability of the instrument to evaluate the execution of forward and backward rolls (Information Scale for Agility on the Soil, InfoSAS) and its suitability to be used by both generalist and specialist professionals to evaluate forward and backward rolls was assessed in a first study, while a second study involving two groups of young children served to confirm the responsiveness of the InfoSAS.

### 2.1. The Instrument InfoSAS

**Figure A3:**
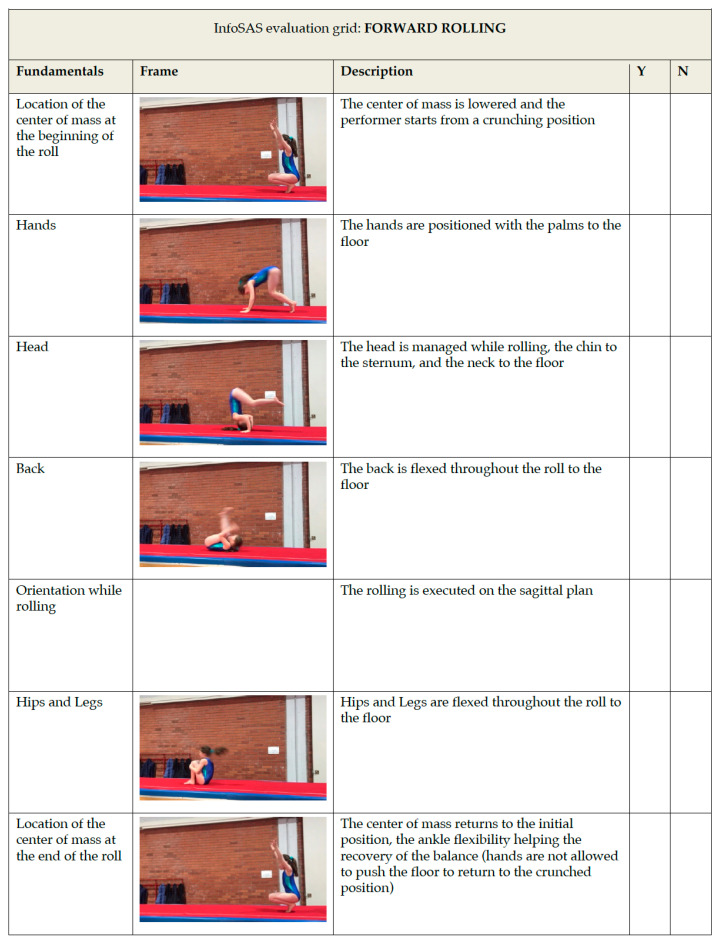
InfoSAS evaluation grid for forward rolling rating.

**Figure A4:**
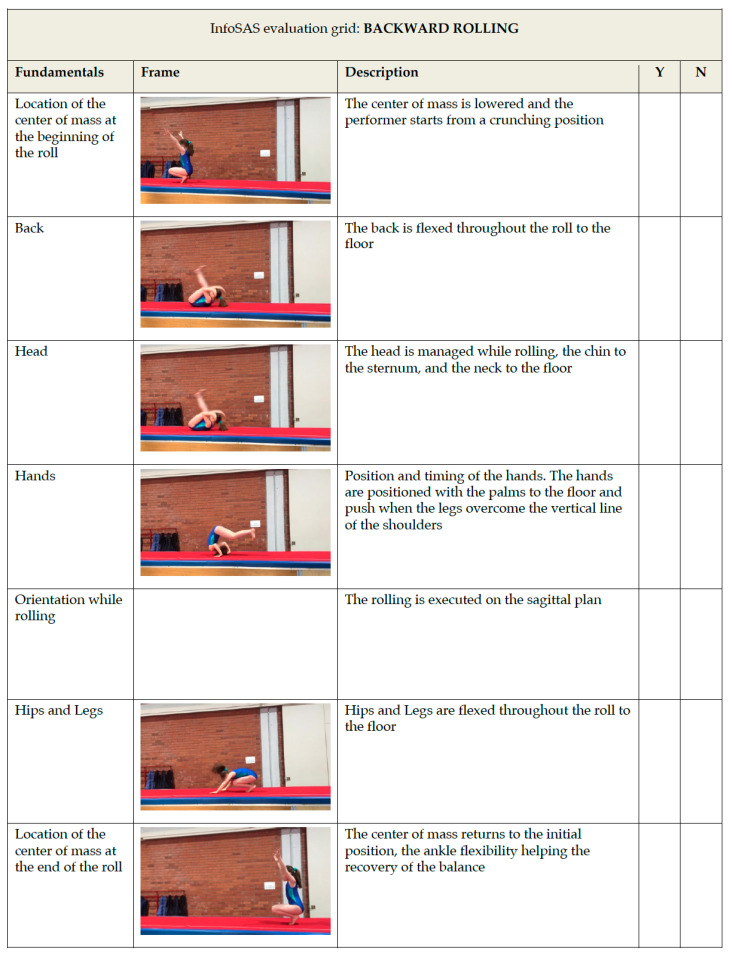
InfoSAS evaluation grid for backward rolling rating.

The instrument InfoSAS to evaluate the execution of forward and backward rolls was arranged by two researchers in exercise sciences and sport. The InfoSAS considers seven fundamentals, attitudes, and skills (CMB = location of the center of mass at the beginning of the roll; HA = hands; HE = head; BA = back; OR = orientation while rolling; HL = hip/legs; and CME = location of the center of mass at the end of the roll) that performers have to manage and successfully achieve while doing rolls (Appendix B, Figure A3 and Figure A4). The performance is scored as Y = 1 point (correct/present) or N = 0 points (failed/missing), the sum of which results in a final score ranging from 0 to 7. Two expert gymnastic instructors approved the logic validity of the instrument and its appropriateness to detect, consider, and discriminate all components of the rolling tasks, and they agreed it can be used to carefully observe and evaluate the execution of the rolling tasks.

The reliability and responsiveness of the InfoSAS and its application by specialist and generalist teachers is one of the goals of this study addressed at teachers’ education.

### 2.2. Study 1—Intra- and Inter-Rater Reliability of the InfoSAS

#### 2.2.1. Participants of Study 1

Five specialists (PE teachers, SPE) and five generalists (teachers of primary school, GEN) participated in this study. SPE were female students (age 23.8 ± 0.8 years) of the last year of the Master’s degree program in Individual and Team Sport Science at the University of Milan. Inclusion criteria were the possession of a Bachelor’s degree in Sport Sciences and no previous experience in PE teaching in primary school. GEN were female teachers (age 42.0 ± 2.5 years) of the public primary school. Inclusion criteria were a minimum of fifteen years of teaching experience and solid knowledge in didactics, teaching, and evaluation, including PE. However, GEN had poorer individual experience in PE than SPE, as their education programs spent a limited amount of time in practical experience compared to the education programs followed by SPE, in which methodologies, productive practice, laboratory involvements in specific training on motor control, and motor learning are integrated with more specific knowledge and competencies in exercise science (Appendix A). All participants signed informed consent prior to beginning the study.

#### 2.2.2. Procedure of Study 1

Video recordings from 13 primary school children (age 6 ± 0.7 years, height 1.18 ± 0.06 m, weight 22.7 ± 3.7 kg, BMI 16.2 ± 1.69 kg/m^2^) acting as models to make a data bank of rolling videos served to investigate the intra- and inter-rater reliability of the InfoSAS and to define whether it can be successfully used by both SPE and GEN.

The participants watched the video recordings of the children’s forward and backward rolls, presented in a random order, and scored them by filling out the InfoSAS. In total, the procedure was repeated three times in three different days to avoid any memory effect.

To better understand how SPE and GEN respectively applied the InfoSAS and in what ways they eventually differed, anthropometrics (BMI) and some parameters defining the flexibility of the children were measured and correlated to the scores assigned by SPE and GEN. Studies have pointed out that, in early childhood, body composition and mobility significantly affect the acquisition of the FMS and, as a consequence, the overpassing of the proficiency barrier [39,40]. Therefore, sit and reach (S&R) and deep squat (DS) tests were collected according to the procedures defined by the literature [41,42].

#### 2.2.3. Statistical Analysis of Study 1

All of the statistical procedures were performed using SPSS (version 20.0 Chicago, IL, USA). The Shapiro–Wilk test revealed that the assumption of normal distribution of each set of data used was not met. To assess the intra-rater reliability for total scores of SPE and GEN, the non-parametric Friedman test with Dunn’s post hoc was applied, whether or not multiple comparisons were necessary, for both forward and backward roll. Similarly, the inter-rater reliability of the total scores was analyzed by applying the Kruskal–Wallis test with Dunn’s post hoc test. Linear regression analysis with Pearson’s coefficient was applied to analyze the correlations of SPE and GEN scores with children’s anthropometrics and flexibility. Means of the raters’ evaluations were used. The alpha value was set at a significance level of 0.05.

### 2.3. Study 2—Responsiveness of the InfoSAS

From study 1, the InfoSAS resulted in being fully reliable only if administered by SPE. Therefore, we decided to complete the research with a second study to further investigate the responsiveness of the InfoSAS to discriminate the forward and backward rolls in children of different skill levels or to discriminate improvements resulting from a period of specific training in rolling. Therefore, three specialists from study 1 were further involved in this second study: two with the role of raters and one as a teacher to conduct the practice of the participants.

#### 2.3.1. Participants of Study 2

Nineteen young children from a primary school (PRI; males: n = 10, females: n = 9; age 6 ± 1 years, height 1.23 ± 0.04 m, weight 22.2 ± 4.0 kg, BMI 14.5 ± 1.70 kg/m^2^) and 12 female gymnasts from a one-year preparatory course practicing gymnastics two days per week (GYM, age 6 ± 1 years, height 1.13 ± 0.06 m, weight 19.3 ± 2.3 kg, BMI 15.2 ± 1.60 kg/m^2^) participated in the study. Inclusion criteria were the absence of pathologies or injuries of the locomotor system, no previous experience in gymnastics for PRI, and more than one year of previous experience in gymnastics for GYM.

#### 2.3.2. Procedure of Study 2

After a brief warm-up, a video of a skilled young competitive gymnast performing a forward and backward roll was shown to the participants. They were asked to reproduce both tasks at their best on a mat sized 1 × 2 × 0.05 m. Two cameras were placed on the front and on the side of the performer at a distance of about 3 m from the mat to record the whole routine for being scored afterward by the InfoSAS.

The video recordings were scored by the raters with the InfoSAS and served to accomplish the first investigation, i.e., to assess whether the instrument is responsive to skill levels. Ratings were performed in blind conditions, i.e., with the skill level of the participants unknown. Because of the previous sport-specific experience, GYM was supposed to be already skilled in rolling, while it was questionable that PRI had comparable abilities. Different scores between them were therefore expected.

Then, PRI attended specific training in rolling at the end of 10 consecutive lessons of physical education at school. The 10-min sessions were led by the specialist (teacher) twice a week, and the children were taught in rolls for a total of 100 min [43]. They underwent the testing protocol once more, which was further scored by the raters to assess the responsiveness of the InfoSAS in detecting changes induced by the brief period of training (post) compared to the status before being taught in rolls (pre). To check the reliability of the rolling, PRI pre-performed three rolls. Since the reliability was confirmed, we considered the best of the three measures to be compared to the scores of PRI-post and GYM, who were tested in forward and backward rolls only once.

#### 2.3.3. Statistical Analysis of Study 2

The Shapiro–Wilk test was applied to test the assumption of normal distribution of each set of data. As the assumption was not met, non-parametric statistics were applied. The reliability of total scores (TS) of PRI and GYM in both forward and backward rolls was assessed by the Friedman test with Dunn’s post hoc for multiple comparisons. To detect the effects of the teaching program on forward and backward rolls, the best TS of PRI before treatment was compared with TS post-training with the Wilcoxon test, whereas partial scores (i.e., the scores of each of the seven fundamentals, attitudes, and skills—CMB, HA, HE, BA, OR, HL, and CME) were analyzed by applying the McNemar test. To compare GYM to PRI pre- and PRI post-TS, the Mann–Whitney U-test was applied, and partial scores were compared with the McNemar test. Further comparisons between GYM, PRI pre, and PRI post forward and backward roll TS were performed by applying the Mann–Whitney test. The alpha value for all performed analyses was set at 0.05.

## 3. Results

### 3.1. Results of Study 1 (Intra- and Inter-Rater Reliability of the InfoSAS)

The results of the intra-rater reliability are presented in Table 1. As SPE comparatively scored in the repeated evaluations, the intra-rater reliability was confirmed in both forward and backward rolling (*p* > 0.05), and the same was for GEN, but only in backward rolling (*p* > 0.05). Conversely, the reliability of GEN in scoring the forward rolls was not confirmed because of the significant difference found among evaluations (*p* < 0.05) despite the fact that post hoc multiple comparisons did not differ.

The inter-rater reliability of the InfoSAS (Table 2) was fully confirmed in SPE. In both forward and backward rolling, all SPE evaluators scored similarly (*p* > 0.05). Differently than SPE, GEN exhibited reliable scorings only in front rolling evaluations (*p* > 0.05), while backward rolling was differently observed among them (*p* < 0.05). In particular, the post hoc comparisons revealed differences in scores between raters #2 and 4, and between raters #3 and 4 (*p* < 0.05).

The scores of forward and backward rolling, as rated with InfoSAS application, did not correlate (*p* > 0.05) with BMI nor with S&R in both SPE and GEN. Similarly, SPE and GEN scores of the forward rolling did not correlate with DS (*p* > 0.05), which, on the contrary, correlated with SPE ratings of backward rolling (R = 0.43, *p* < 0.05), and did not correlate with GEN ratings (*p* > 0.05).

### 3.2. Results of Study 2 (Responsiveness of the InfoSAS)

Statistics confirmed the reliability of TS in both forward and backward roll (Forward roll PRI: #1 = 3.5 ± 1.6 AU, #2 = 3.7 ± 1.5 AU, #3 = 3.6 ± 1.6 AU, *p* = 0.074; Forward roll GYM: #1 = 6.8 ± 0.4 AU, #2 = 6.8 ± 0.6 AU, #3 = 6.7 ± 0.8, *p* = 0.223; Backward roll PRI: #1 = 2.3 ± 1.7 AU, #2 = 2.3 ± 1.5 AU, #3 = 2.4 ± 1.6 AU, *p* = 0.368; Backward roll GYM; #1 = 4.5 ± 2.4 AU, #2 = 4.5 ± 2.5 AU, #3 = 4.5 ± 2.5 AU, *p* = 1.000).

Table 3 synthesizes all comparisons that served to define the responsiveness of the InfoSAS in detecting any difference between expertise levels or improvements after specific training. From TS comparisons, GYM was confirmed to perform forward rolls better than both PRI pre and PRI post (6.8 ± 0.4 vs. 3.7 ± 1.5, and 5.0 ± 1.6; points; *p* < 0.05), and backward rolling better than PRI pre (4.5 ± 2.5 vs. 2.4 ± 1.6; points; *p* < 0.05). After training, PRI markedly increased TS in both forward and backward rolling (3.7 ± 1.5 vs. 5.0 ± 1.6 points; 2.4 ± 1.6 vs. 4.0 ± 2.1, respectively; *p* < 0.05). PRI also improved with training, and reached comparable results to GYM in backward rolling (4.0 ± 2.1 vs. 4.5 ± 2.5 points, *p* > 0.05).

The partial scores analysis (Table 3, bottom half) highlighted whether, with training, some features more than others contributed in making the difference between TS. To be noticed, BA and OR appeared to be the main source of improvements in all rolling conditions (forward rolling: *p* = 0.016 and *p* = 0.016, BA and OR, respectively; backward rolling: *p* = 0.008 and *p* = 0.008, BA and OR, respectively). HL further contributed to backward rolling improvements (*p* = 0.031). Markedly, looking at the forward rolling changes in the seven fundamentals, attitudes, and skills of PRI (which also reflect the changes in scores due to the training effect), PRI pre differed from GYM in BA, OR, HL, and CME (*p* = 0.008, 0.004, 0.002, and 0.012, respectively), while PRI post almost reached GYM and only differed in HL and CME (*p* = 0.016, and 0.039, respectively), which highlights and supports the positive effects the training had on PRI.

**Figure 1:**
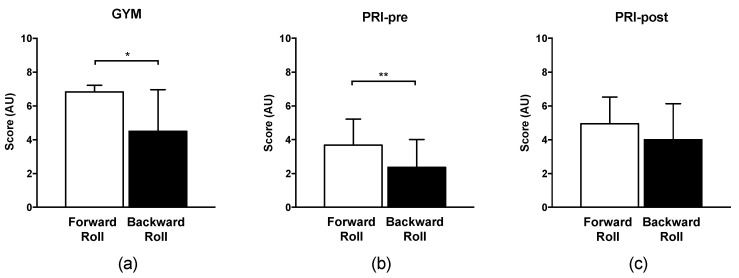
Comparisons between forward and backward rolling in GYM (**a**) PRI pre-training (**b**) and PRI post-training (**c**). PRI = primary school children; GYM = gymnasts. * = *p* < 0.05; ** = *p* < 0.01.

Figure 1 shows the comparisons between the front and backward rolling TS for each condition. The training seems to have been extremely beneficial to backward rolling, which scored similarly to forward rolling in PRI post (Figure 1, Panel c; *p* > 0.05), whereas in both GYM and PRI pre, forward rolling was more highly scored than the backward one (Figure 1, Panel a and b, respectively; *p* < 0.05).

## 4. Discussion

This explorative study was designed to investigate the reliability and responsiveness of the instrument InfoSAS to evaluate the execution of the forward and backward roll (agility on the soil) in young children, and to further verify its suitability and application by specialist or generalist teachers by comparing their proficiency in assessing the rolling motor task. From the results of study 1, the InfoSAS appeared to be reliable and therefore suitable to be administered only by specialists, hardly applicable by generalists. The second study, which was consequently carried out only with specialists’ support, confirmed that the InfoSAS was responsive in detecting differences due to expertise (gymnasts vs. beginners) or due to improvements resulting from specific training in rolling.

The results of this study preliminarily suggest that: (i) the instrument is reliable and responsive to evaluate forward and backward rolling in children; (ii) being the instrument not reliable with generalists, it seems to require specific competence to be administered, and appears to be suitable only for specialists; and (iii) specialists and generalists highly differ in assessing the rolling abilities with the InfoSAS.

### 4.1. Study 1 (Intra- and Inter-Rater Reliability of the InfoSAS)

The intra-rater analysis (Table 1) confirmed that SPE was reliable in repeated observations, unlike GEN, which did not consistently rate the participants’ forward roll performances. Similarly, the inter-rater analysis highlighted that all SPE raters scored comparably, while GEN’s evaluations differed between them in judging the backward rolling. Altogether, the results support that GEN is not as reliable as SPE in InfoSAS application, which seems to be suitable for use only by SPE.

Even if the InfoSAS thoroughly defines the procedure to evaluate the rolling performance, GEN hardly detected and realized all specifics of the motor tasks’ accomplishment, possibly because of the reduced individual experience in physical activity compared to SPE. Differently, SPE was confirmed to properly focus on all of the performers’ actions that are important to be observed and scored (and managed while teaching). The literature reported frequent use of thinking shortcuts when the resources to process information are limited, as in the case of generalist teachers without specific individual practical experience [44]. Due to this phenomenon, individuals do not analytically and precisely consider all given information, which can result in alterations and reasoning errors. Perception is a constructive process whose results are filtered by interpretation, which is activated by the brain and which depends on individual practice and personal emotional experiences [45,46]. Sport Science graduates, thanks to the adequate education and corporal consciousness they practiced along with their studies, have an “experiential track” representing perceptive and interpretative filtering of physical, psycho-social, emotional, and experiential memories.

These memories from individual experiences are essential to expanding the conceptualization and the interpretation of situational constraints (e.g., the evaluation of a motor task through the application of the InfoSAS, as in our case), distinguishing the daily professional activity of PE teachers [47,48,49]. Further sources of knowledge, such as skills observation training by sheet support (i.e., laboratory experiences included in the PE studies curricula), also enhance competence in objective evaluations according to the conceptual category’s model [50,51,52].

Competence is the ability to correlate the “old to the new”, that is, to identify similarities between actual and previously solved problems, which enlarges the collection of mental representations at the disposal of competent experts to deal with specific situations in a wide range of resolutions and by most effective actions [53]. Recognizing the movement patterns is the key point of problem-solving skills, which allows categorizing new occurrences in classes of events already familiar [54]. Memories from knowledge, skills, and perception–action processes by the emotional, executive, and social intelligences allow the specialist to make decisions that are the result of a post-analytical synthesis of previous experiences (Appendix A, Figure A2).

The learning process can be enhanced and promoted by widening and connecting physical, motor, social, and cognitive experiences among them [55]. Through the practices of verbalization with peers and teachers, and by applying specific tools such as “the evaluation wheel”, an inner reflection process can be improved. Then, psychological constructs, such as attention, memory, abstract concepts, critical thinking, and socio-emotional development can be built to create meanings and awareness [56] to better understand and interpret physical literacy at any level [57]. Lived practice in gyms generates emotional experiences that are the rudder that directs thinking and helps information recall of relevant issues to be used to deal with problems [51].

Former learnings, attitudes, predispositions, and current contexts strongly affect the discernment of reality and how it is interpreted [50,58]. Action reading never corresponds to objective evaluations, rather, it depends on personal constructs coming from individual perceptions, emotions, and experiences. Evidence from affective neuroscience connects body and mind in the emotional processes [58], and evidence from social neuroscience empathetically connects the individuals, the teacher to the learner in particular [51,59]. Specific features, such as analytical deepening, motor practice based on reflexive approaches, and cooperative learning, characterize the great difference between specialists and generalists [58,60] while leading PE classes in the primary school [13] and while properly observing, interpreting, and conducting diagnostics and evaluations of the movements’ outputs, as in the case of our study 1, which contemplated the rolling patterns.

Lastly, the higher ability of SPE in evaluating the rolling abilities compared to GEN is further supported by the correlation that has been found in backward rolling between TS and DS, which was retrieved only in SPE. Therefore, the evaluation of backward rolling given by SPE seems to be somehow linked to DS. That can possibly be explained by ankle flexibility, which is particularly relevant in DS testing, and whose observation while scoring the backward rolling requires a level of knowledge, competence, and experience not familiar to and not possessed by GEN.

Indeed, the DS testing procedure allows measurement of the flexibility of the back, knee, and ankle. These components have been confirmed to be fundamental to hold the right attitude while rolling. In particular, ankle flexibility is important in the final phase of backward rolling, when the performer has to return to the standing position from its lower point, having the hip and knees in their maximal bending [61]. Contrary to front rolling, the ankle dorsiflexes in the final phase of backward rolling from the extended position, and the correct execution of this rolling requires the performer to be more skilled than needed on front rolling. At the same time, the observer (and rater) of the rolling outcome needs to be particularly sensitive in reading and detecting the skills of this last part of execution, and in judging their contribution to the success of the whole rolling action. Possibly because of their poorer individual practical proficiency, GEN failed in this task-reading, while, thanks to individual previous experience, SPE differentiated from GEN in the fine reading of ankle flexibility and its contribution to the task required. As a consequence, TS might have reflected this sensitivity and consequently correlated to DS as a measure of flexibility. The InfoSAS carefully describes all phases and features of the forward and backward rolling, but is presumably sufficiently accurate and user friendly only for specialists to ensure good measurements and assessments of rolling abilities through an easy form [62]. This further supports that the InfoSAS is reliable only when administered by SPE.

### 4.2. Study 2 (Responsiveness of the InfoSAS)

The responsiveness of the InfoSAS and its logic validity that make it applicable by SPE to detect and discriminate the differences of rolling actions due to expertise or training effects were confirmed based on the results from Study 2 (Table 2).

Concerning expertise, the young female gymnasts composing the GYM group had at least one year of practice in gymnastics skills, while PRI were children attending the first grade of primary school and had non-specific motor experience coming from different sports practice. The results from the use of the InfoSAS by the specialists agree with the literature: gymnastics practice improved the skills required to perform the rolling more than other motor activities [63]. In particular, in the forward rolling, the gap between GYM and PRI appeared to be more evident in the central and final phases of the rolling (Table 2).

This might depend on the specific expertise and practice of GYM in managing body control throughout the whole action because of the specific features of the sport, and because of the need to hold complete control until the end of the movement to be ready to continue in successive figures, which results in better agility on the soil of GYM than PRI. The development of motor abilities is age-related and not age-dependent [64]. In our case, GYM had previously practiced more structured training in rolls than PRI, which lacked any specific guided practice and could not have developed comparable levels of motor abilities by themselves. In the backward rolling, even if GYM was confirmed to score better than PRI before the training period of rolling, they did not differ from PRI after training. This seems to suggest that while GYM is more skilled than PRI, its limited practice (one year only) diminished the differences post-training from the non-expert children and allowed PRI to approach GYM in performing the backward rolling, a more complex skill than the forward roll. This agrees with Brian et al. [23] and confirms that the transition from a rudimental skill level to an advanced one requires more practice and makes the proficiency barrier a difficult goal to overcome without an adequate period of specific and structured practice.

Concerning the training effects, the comparisons of pre- and post-training scores (Table 2) also confirmed our hypothesis that the InfoSAS is suitable to detect improvements of rolling abilities when non-experts are taught by specific activities, even if for a relatively short time and volume (10 min in 10 consecutive lessons). In studies on falling ability, DelCastillo-Andres, et al. [43] and Invernizzi, et al. [65] used comparable amounts of practice to train rolling participants from primary and secondary school, respectively. Moreover, BA and OR fundamental skill scores highlight the higher benefits resulting from training sessions in both forward and backward rolling. As BA and OR refer to the back and the orientation administration, that is, the main phase on the soil while rolling, this also suggests that the InfoSAS can be applied to return information about agility on the soil.

Finally, from the results (Figure 1, panels a and b), the forward rolling was confirmed to be easier perform than the backward rolling in both GYM and PRI, and the InfoSAS did not fail in detecting that. However, as PRI in the post-training scored comparably in both rolling directions, brief specific training seems to be successful in reducing the abovementioned gap (Figure 1, panel c). Because of requirements of accurate muscular activity, timing, and coordination, the backward roll has been reported to be quite difficult to perform. Conversely, for the same reason, it is widely used by PE teachers to improve the movement abilities of children [66]. The comparable results of forward and backward rolling after training of PRI further imply some effects on learning from the age of the participants (six years), which must be considered. According to Meijer and Roth [67], even depending on several factors that produce a wide variability in learning and performing, the sensitive period to learn the skills for the backward roll is set at five to six years of age, which was the same age as the participants of our study. Differently, the skills required by forward rolling are acquired a bit earlier [68], which possibly explains why the scores of the forward roll are so high compared to those of the backward roll, and why the improvements of the latter are superior to those of the forward roll.

### 4.3. Authentic Tasks and Competences

In the didactics, an instrument such as the InfoSAS can be defined as an authentic task [69]. Authentic tasks aim to replicate as far as possible the actual circumstances in which the performer (in our case, the teachers) has to prove his knowledge and ability to apply the previously acquired didactic skills to solve any challenge related to the assignment addressed (problem-solving in the teaching process). Thanks to authentic tasks, the teacher engages autonomy and responsibility; he activates to build his knowledge, to select and decide, and to be responsible for his actions and subsequent implications on learners [70,71].

Biggs and Tang [72] pointed out the key points to be followed in academic education to maximize the final result of the education process at the university. In particular, they highlighted how the final results of the learning process have to be depicted as competencies to be acquired that embrace combinations of cognitive and metacognitive abilities, knowledge and understanding, and interpersonal, intellective, and practical attitudes.

Competence levels can be verified exclusively in situational conditions, such as in our studies 1 and 2. Indeed, competence is the ability to apply decision-making and properly operate and react to the specific and actual conditions that occur.

Therefore, competence can be specifically assessed by means of reality tasks [69]. Morin [73] pointed out the concept of education to the complexity, and how teaching should make transferable competences to the social reality of which anyone is part, as well as how the methodology is particularly relevant to this purpose. Experiential approaches are based on reflection, as in the specialists’ specific education, and focus on making autonomy, competence, and relationships perfectly fit.

The differences between specialists’ and generalists’ outcomes, as resulting from our study, seem to confirm some insights from the literature. Vygotskij [74] highlighted that the focus of the task (the teaching process, in this case) should be set in a zone of proximal personal development in which the scenario is not well-known, but can be properly addressed and managed given that any required cognitive and operative tools (high specific competencies) are possessed already, as in the case of the specialists of our study. As a result, practitioners taught by specialists exhibit higher levels of motor skills than practitioners taught by generalists [75]. Generalists seem mainly to offer to the practitioners free play-based activities, while specialists widely and specifically address the focus of the activities they propose on the development of motor skills and abilities, being more competent and used to qualitatively observe and conduct the practice than non-specialists [76]. Competent teachers merely enforce the promotion of motor competencies and related assessments to stimulate health prevention, and active and healthy lifestyles of practitioners by a properly oriented leading of physical activity.

## 5. Conclusions

Our preliminary results suggest that specialists and generalists highly differ in the assessment of rolling abilities by applying the InfoSAS evaluation grid, possibly because of the different levels of competence and individual practice they previously experienced along with their academic education.

The InfoSAS has been proved to be reliable and responsive in evaluating the rolling abilities of children in primary school. The InfoSAS can be applied by specialists who have competence in sport sciences and have previous experience of individual practice and teaching styles in physical activity, while generalist teachers failed to satisfactorily apply this instrument of evaluation. The specialists can apply this evaluating tool to properly and adequately judge the performances of the learners and to discriminate the outcomes in rolling abilities depending on expertise or on the effects of training the skills involved in rolling, even if lasting for a limited amount of time. The InfoSAS can also be applied to focus on skills defining agility on the soil.

Application and validation of the reliability and responsiveness of the InfoSAS with older children are advisable, as well as investigations on the retention after training sessions and on the effects of specific additional exercises to improve ankle flexibility. Further research should also be aimed at comparing specialists and generalists in applying alternative tools to evaluate motor patterns other than rolling. It must be said that a larger sample size could be useful to reach the normal distribution of the data and to deepen and possibly confirm the results of the present exploring study. A further and not trivial limitation of the present research is represented by the lack of a control group in study 2; the presence of a control group could have been very useful to rule out that an improvement in the two experimental conditions had been by chance.

From the results of our study, we can extend our assumptions to the needs of the teaching process of physical activity, and we can conclude that PE in primary school should be necessarily guided by competent professionals in sport sciences. In addition, this study exemplifies how the research itself might serve to assess and review some specific targets of academic education in sports sciences. This process can help institutions to find strengths or weaknesses of the curricula, to check whether their aims and teaching styles adequately succeed in making proficient professionals not exclusively in knowledge but in competences as well, and to make proficient specialists in assessing, judging, and managing the widest spectrum of motor patterns and skills.

## Figures and Tables

**Table 1:** Intra-rater reliability of the InfoSAS.

	Rolling	Score #1 (AU)	Score #2 (AU)	Score #3 (AU)	*p*-Value	Post Hoc #1 vs. #2	Post Hoc #2 vs. #3	Post Hoc #1 vs. #3
SPE	Forward	3.3 ± 1.7	3.4 ± 2.2	3.7 ± 2.3	0.087			
	Backward	1.1 ± 1.5	1.1 ± 1.9	1.1 ± 2.1	0.926			
GEN	Forward	5.1 ± 1.8	4.5 ± 2.4	5.4 ± 1.8	0.024 *	0.233	0.075	1.00
	Backward	3.0 ± 2.6	2.9 ± 2.5	2.4 ± 2.4	0.157			

Scores are expressed as mean ± SD. SPE = specialists; GEN = generalists. *p*-values refer to the Friedman non-parametric test, with Dunn’s post hoc test for multiple comparisons. * = *p* < 0.05.

**Table 2:** Inter-rater reliability of the InfoSAS.

	Rolling	Rater #1	Rater #2	Rater #3	Rater #4	Rater #5	*p*	Post Hoc
SPE	Forward	2.2 ± 1.7	2.2 ± 1.7	2.1 ± 2.3	2.1 ± 2.1	1.8 ± 1.9	0.908	
	Backward	0.5 ± 1.1	0.5 ± 1.1	0.6 ± 1.7	0.5 ± 1.7	0.6 ± 1.2	0.910	
GEN	Forward	4.2 ± 2.8	2.1 ± 1.7	2.8 ± 2.5	3.5 ± 2.1	2.8 ± 2.2	0.206	
	Backward	1.7 ± 2.7	0.8 ± 1.4	0.8 ± 2.1	2.8 ± 1.9	1.5 ± 1.0	0.002 *	#3 vs. #4: 0.0R04 * #2 vs. #4: 0.0R26 *

Scores are expressed as mean ± SD. SPE = specialists; GEN = generalists. * = *p* < 0.05.

**Table 3:** Comparisons between pre- and post-training and between PRI (primary school children) and GYM (gymnasts).

Rolling		% of Success of the Partial Scores (100% = 7 pts)							Scores (pts)
		CMB	HA	HE	BA	OR	HL	CME	TS
Forward	PRI pre	94.7	94.7	94.7	36.8	31.6	10.5	5.3	3.7 ± 1.5
	PRI post	100.0	100.0	94.7	73.7	68.4	36.8	21.1	5.0 ± 1.6
	GYM	100.0	100.0	100.0	100.0	100.0	100.0	83.3	6.8 ± 0.4
Backward	PRI pre	94.7	73.7	21.1	15.8	10.5	15.8	5.3	2.4 ± 1.6
	PRI post	100.0	89.5	47.4	57.9	52.6	47.4	5.3	4.0 ± 2.1
	GYM	100.0	100.0	58.3	50.0	50.0	50.0	41.7	4.5 ± 2.5
Forward	PRI pre vs. PRI post	1.000	1.000	1.000	0.016 *	0.016 *	0.063	0.250	0.005 **
	PRI pre vs. GYM	1.000	1.000	1.000	0.008 **	0.004 **	0.002 **	0.012 *	0.003 **
	PRI post vs. GYM	1.000	1.000	1.000	0.250	0.125	0.016 *	0.039 *	0.009 **
Backward	PRI pre vs. PRI post	1.000	0.250	0.063	0.008 **	0.008 **	0.031 *	1.000	0.001 **
	PRI pre vs. GYM	1.000	0.125	0.219	0.125	0.125	0.125	0.063	0.016 *
	PRI post vs. GYM	1.000	0.500	1.000	1.000	1.000	1.000	0.063	0.447

Scores are expressed as a % of success in the partial scores (100% = 7 pts) and as mean ± SD in the total scores (TS). CMB = location of the center of mass at the beginning of the roll; HA = hands; HE = head; BA = back; OR = orientation while rolling; HL = hips/legs; CME = location of the center of mass at the end of the roll. * = *p* < 0.05; ** = *p* < 0.01.

## References

[1] Gazzetta Ufficiale della Repubblica Italiana (2013). Definizione delle norme generali e dei livelli essenziali delle prestazioni per l’individuazione e validazione degli apprendimenti non formali e informali e degli standard minimi di servizio del sistema nazionale di certificazione delle competenze. Gazzetta Ufficiale della Repubblica Italiana.

[2] D’Isanto T. (2019). Physical and sport education between Italian academic system and European Research Council structure panel. J. Hum. Sport Exerc..

[3] D’Isanto T. (2019). State of art and didactics opportunities of Physical Education teaching in Primary School. JPES.

[4] Ray J.D., Overman A.S. (2014). Hard Facts About Soft Skills. Am. J. Nurs..

[5] Official Journal of the European Union (2018). Council recommendation of 22 May 2018 on key competences for lifelong learning. Off. J. Eur. Union.

[6] Gazzetta Ufficiale della Repubblica Italiana (2007). Regolamento recante norme in materia di adempimento dell’obbligo di istruzione. Gazzetta Ufficiale della Repubblica Italiana.

[7] D’Elia F., Raiola G. The core curriculum in the university training of the teacher of physical education in Italy. Proceedings of the Journal of Human Sport and Exercise—2018—Spring Conferences of Sports Science.

[8] De Meester A., Stodden D., Goodway J., True L., Brian A., Ferkel R., Haerens L. (2018). Identifying a motor proficiency barrier for meeting physical activity guidelines in children. J. Sci. Med. Sport.

[9] Orth D., van der Kamp J., Memmert D., Savelsbergh G.J.P. (2017). Creative Motor Actions As Emerging from Movement Variability. Front. Psychol..

[10] MacPhail A., Halbert J. (2010). ‘We had to do intelligent thinking during recent PE’: Students’ and teachers’ experiences of assessment for learning in post-primary physical education. Assess. Educ..

[11] Dyson B., Grineski S. (2001). Using Cooperative Learning Structures in Physical Education. JOPERD.

[12] Posner M.I., Rothbart M.K. (2007). Research on attention networks as a model for the integration of psychological science. Annu. Rev. Psychol..

[13] Invernizzi P.L., Crotti M., Bosio A., Cavaggioni L., Alberti G., Scurati R. (2019). Multi-Teaching Styles Approach and Active Reflection: Effectiveness in Improving Fitness Level, Motor Competence, Enjoyment, Amount of Physical Activity, and Effects on the Perception of Physical Education Lessons in Primary School Children. Sustainability.

[14] Glasser W. (1999). Choice Theory: A New Psychology of Personal Freedom.

[15] Coelho J. (2010). Gymnastics and Movement Instruction Fighting the Decline in Motor Fitness. JOPERD.

[16] Možnik M., Milčić L., Živčić Marković K. Motor Knowledge and Process of Learning Basic Gymnastic Elements in Students of Faculty of Kinesiology. Proceedings of the 6th International Scientific Conference.

[17] Rudd J.R., Barnett L.M., Butson M.L., Farrow D., Berry J., Polman R.C. (2015). Fundamental Movement Skills Are More than Run, Throw and Catch: The Role of Stability Skills. PLoS ONE.

[18] Haywood K., Getchell N. (2014). Life Span Motor Development.

[19] Langendorfer S.J., Roberton M.A. (2002). Individual pathways in the development of forceful throwing. Res. Q. Exerc. Sport.

[20] Wickstrom R.L. (1977). Fundamental Motor Patterns.

[21] Claxton D.B., Troy M., Dupree S. (2006). A Question of Balance. JOPERD.

[22] Seefeldt V., Nadeau C., Holliwell W., Roberts G. (1980). Developmental motor patterns: Implications for elementary school physical education. Psychology of Motor Behavior and Sport.

[23] Brian A., Getchell N., True L., De Meester A., Stodden D.F. (2020). Reconceptualizing and Operationalizing Seefeldt’s Proficiency Barrier: Applications and Future Directions. Sports Med..

[24] Malina R.M. (2014). Top 10 research questions related to growth and maturation of relevance to physical activity, performance, and fitness. Res. Q. Exerc. Sport.

[25] Magill R., Smoll F.L., Magill R.A., Ash M.J. (1988). Critical Periods of Optimal Readiness Learning Sports Skill. Children in Sport.

[26] Logan S.W., Webster E.K., Getchell N., Pfeiffer K.A., Robinson L.E. (2015). Relationship Between Fundamental Motor Skill Competence and Physical Activity During Childhood and Adolescence: A Systematic Review. Kinesiol. Rev..

[27] Dumith S.C., Ramires V.L.V., Souza M.A., Moraes D.S., Petry F.C.G., Oliveira E.S., Ramires S.V., Hallal P.C. (2000). Overweight/Obesity and Physical Fitness Among Children and Adolescents. J. Phys. Act. Health.

[28] Williams K. (1980). Developmental characteristics of a forward roll. Res. Q. Exerc. Sport.

[29] Syahruddin S., Latuheru R.V. (2019). The Effect of The Strength of Extremity and Motivation on Forward Roll of Achievement Learning. J. Phys. Educ. Sport.

[30] Sacchetti R., Ceciliani A., Garulli A., Masotti A., Poletti G., Beltrami P., Leoni E. (2012). Physical fitness of primary school children in relation to overweight prevalence and physical activity habits. J. Sports Sci..

[31] Fang H., Quan M., Zhou T., Sun S., Zhang J., Zhang H., Cao Z., Zhao G., Wang R., Chen P. (2017). Relationship between Physical Activity and Physical Fitness in Preschool Children: A Cross-Sectional Study. Biomed. Res. Int..

[32] De Meester A., Maes J., Stodden D., Cardon G., Goodway J., Lenoir M., Haerens L. (2016). Identifying profiles of actual and perceived motor competence among adolescents: Associations with motivation, physical activity, and sports participation. J. Sports Sci..

[33] DelCastillo-Andres O., Toronjo-Hornillo L., Toronjo-Urquiza M.T., Toronjo-Urquiza L., Campos-Mesa M.d.C., Invernizzi P.L., Genovesi E., Morvay-Sey K., Kerner I., Carlsen H.F. Development and internationalization of proactive programs to teach how to fall: Adapted utilitarian judo and safe fall-safe schools©. Proceedings of the Book of the 6th European Judo Science & Research Symposium and 5th Scientific and Professional Conference.

[34] Kalina R.M., Barczyński B., Jagiełło W., Przeździecki B., Kruszewski A., Harasymowicz J., Syska J., Szamotulska K. (2008). Teaching of safe falling as most effective element of personal injury prevention in people regardless of gender, age and type of body build—The use of advanced information technologies to monitor the effects of education. Arch. Budo.

[35] Novak J.D., Gowin D.B. (2001). Imparando a Imparare.

[36] Bruner J.S. (1957). Neural mechanisms in perception. Psychol. Rev..

[37] Deci E.L., Ryan R.M. (2000). The “What” and “Why” of Goal Pursuits: Human Needs and the Self-Determination of Behavior. Psychol. Inq..

[38] Ntoumanis N. (2005). A Prospective Study of Participation in Optional School Physical Education Using a Self-Determination Theory Framework. J. Educ. Psychol..

[39] Clark J.E. (2005). From the Beginning: A Developmental Perspective on Movement and Mobility. Quest.

[40] Vameghi R., Shams A., Shamsipour Dehkordi P. (2013). The effect of age, sex and obesity on fundamental motor skills among 4 to 6 years-old children. Pak. J. Med. Sci.

[41] Wells K.F., Dillon E.K. (1952). The Sit and Reach—A Test of Back and Leg Flexibility. Res. Q. Am. Assoc. Health.

[42] Frost D.M., Beach T.A.C., Callaghan J.P., McGill S.M. (2012). Using the Functional Movement Screen™ to Evaluate the Effectiveness of Training. J. Strength Cond. Res..

[43] DelCastillo-Andres O., Toronjo-Hornillo L., Rodriguez-Lopez M., Castaneda-Vazquez C., Campos-Mesa M.D.C. (2018). Children’s Improvement of a Motor Response during Backward Falls through the Implementation of a Safe Fall Program. Int. J. Environ. Res. Public Health.

[44] Taylor S.E., Hamilton D.L. (2015). A Categorization Approach to Stereotyping. Cognitive Processes in Stereotyping and Intergroup Behavior.

[45] Ablin J.L. (2008). Learning as Problem Design Versus Problem Solving: Making the Connection Between Cognitive Neuroscience Research and Educational Practice. MBE.

[46] CASEL, University of Illinois at Chicago‘s Collaborative for Academic, Social, and Emotional Learning Social and Emotional Learning (SEL) and Student Benefits: Implications for the Safe Schools/Healthy Students core Elements. https://www.eccnetwork.net/resources/social-emotional-learning-sel-and-student-benefits.

[47] Fiske S.T., Taylor S.E. (1991). Social Cognition.

[48] Kruglanski A.W. (1989). The psychology of being “right”: The problem of accuracy in social perception and cognition. Psychol. Bull..

[49] Markus H. (1977). Self-schemata and processing information about the self. J. Pers. Soc. Psychol..

[50] Immordino-Yang M.H. (2008). The Smoke Around Mirror Neurons: Goals as Sociocultural and Emotional Organizers of Perception and Action in Learning. MBE.

[51] Immordino-Yang M.H., Damasio A. (2007). We Feel, Therefore We Learn: The Relevance of Affective and Social Neuroscience to Education. MBE.

[52] Immordino-Yang M.H., Faeth M., Sousa D.A. (2010). The Role of Emotion and Skilled Intuition in Learning. Mind, Brain, & Education: Neuroscience Implications for the Classroom.

[53] Sternberg R.J. (1985). Implicit theories of intelligence, creativity, and wisdom. J. Pers. Soc. Psychol..

[54] Simon H.A. (1996). Models of My Life.

[55] Nelson D.R., Adger W.N., Brown K. (2007). Adaptation to Environmental Change: Contributions of a Resilience Framework. Annu. Rev. Environ. Resour..

[56] Bruner J.S. (1990). Acts of Meaning.

[57] Whitehead M. (2013). Definition of Physical Literacy and Clarification of Related Issues.

[58] Oberman L.M., Pineda J.A., Ramachandran V.S. (2007). The human mirror neuron system: A link between action observation and social skills. Soc. Cogn. Affect. Neurosci..

[59] Harris A., Jones M. (2010). Professional learning communities and system improvement. Improv. Sch..

[60] Faulkner G.E.J., Dwyer J.J.M., Irving H., Allison K.R., Adlaf E.M., Goodman J. (2008). Specialist or Nonspecialist Physical Education Teachers in Ontario Elementary Schools: Examining Differences in Opportunities for Physical Activity. Alberta J. Educ. Res..

[61] Mochizuki L., Sacco I.C.N., Faro A., Amadio A.C. A biomechanical study of dynamics and EMG in gymnastics: Backward roll to handstand. Proceedings of the 13 International Symposium on Biomechanics in Sports.

[62] Marentič-Požarnik B., Konvalinka K. (2000). Psihologija Učenja in Pouka.

[63] Mellos V., Dallas G., Kirialanis P., Fiorilli G., Di Cagno A. (2014). Comparison between physical conditioning status and improvement in artistic gymnasts and non-athletes peers. Sci. Gymnast. J..

[64] Clark J.E., Dent-Read C., Zukow-Goldring P. (1997). A dynamical systems perspective on the development of complex adaptive skill. Evolving Explanations of Development: Ecological Approaches to Organism-Environment Systems.

[65] Invernizzi P.L., Signorini G., Michielon G., Padulo J., Scurati R. (2019). The “safe falls, safe schools” multicentre international project: Evaluation and analysis of backwards falling ability in Italian secondary schools. JPES.

[66] Kovač M. (2012). Assessment of gymnastic skills at physical education—The case of backward roll. Sci. Gymnast. J..

[67] Meijer O.G., Roth K. (1988). Complex Movement Behaviour. The Motor-Action Controversy.

[68] Roth K. (1982). Strukturanalyse Koordinativer Fähigkeiten [Structure Analysis of Coordination Skills].

[69] Tessaro F. (2014). Authentic tasks or reality tests?. Form. Insegn..

[70] Comoglio M. (2002). La valutazione autentica. Orientam. Pedagog..

[71] Wiggins G. (1998). Educative Assessment. Designing Assessments to Inform and Improve Student Performance.

[72] Biggs J.B., Tang C. (2011). Teaching for Quality Learning at University. What the Student Does.

[73] Morin E. (2000). La Testa ben Fatta. Riforma Dell’insegnamento e Riforma del Pensiero.

[74] Vygotskij L.S. (1992). Pensiero e Linguaggio: Ricerche Psicologiche.

[75] Faucette N., McKenzie T.L., Patterson P. (1990). Descriptive Analysis of Nonspecialist Elementary Physical Education Teachers’ Curricular Choices and Class Organization. J. Teach. Phys. Educ..

[76] Placek J.H., Randall L. (1986). Comparison of Academic Learning Time in Physical Education: Students of Specialists and Nonspecialists. J. Teach. Phys. Educ..

